# Donnan Potential across the Outer Membrane of Gram-Negative Bacteria and Its Effect on the Permeability of Antibiotics

**DOI:** 10.3390/antibiotics10060701

**Published:** 2021-06-11

**Authors:** Olaniyi Alegun, Ankit Pandeya, Jian Cui, Isoiza Ojo, Yinan Wei

**Affiliations:** Department of Chemistry, University of Kentucky, Lexington, KY 40506, USA; Olaniyi.Alegun@uky.edu (O.A.); pandeya.ankit@uky.edu (A.P.); Jian.Cui@uky.edu (J.C.); Isoiza.ojo@uky.edu (I.O.)

**Keywords:** Donnan potential, Gram-negative bacteria, antibiotics, membrane permeability, cellular accumulation

## Abstract

The cell envelope structure of Gram-negative bacteria is unique, composed of two lipid bilayer membranes and an aqueous periplasmic space sandwiched in between. The outer membrane constitutes an extra barrier to limit the exchange of molecules between the cells and the exterior environment. Donnan potential is a membrane potential across the outer membrane, resulted from the selective permeability of the membrane, which plays a pivotal role in the permeability of many antibiotics. In this review, we discussed factors that affect the intensity of the Donnan potential, including the osmotic strength and pH of the external media, the osmoregulated periplasmic glucans trapped in the periplasmic space, and the displacement of cell surface charges. The focus of our discussion is the impact of Donnan potential on the cellular permeability of selected antibiotics including fluoroquinolones, tetracyclines, β-lactams, and trimethoprim.

## 1. The Composition of the Gram-Negative Bacterial Cell Envelope

**Figure 1:**
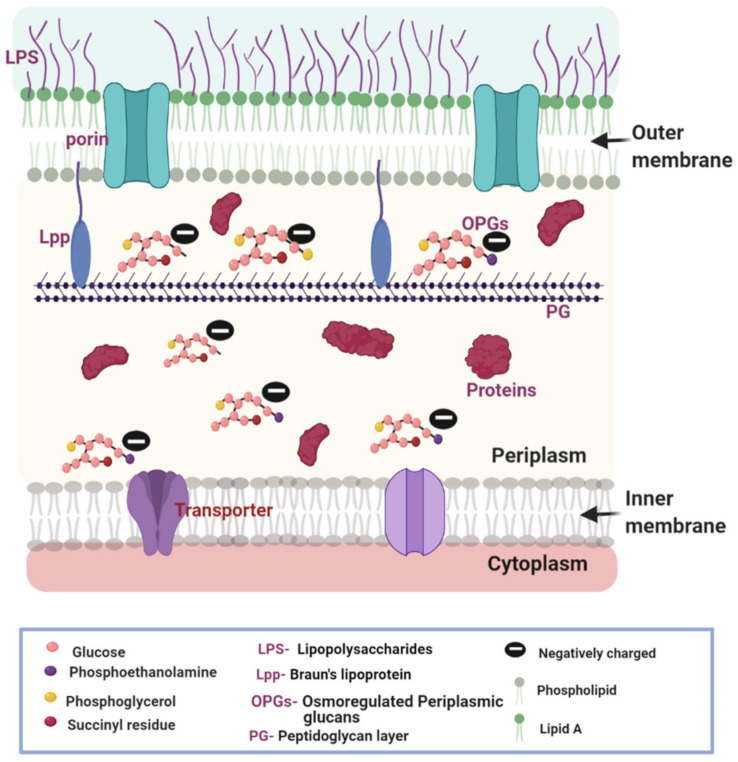
Cell envelope of the Gram-negative bacteria is composed of two layers of lipid bilayer membranes and an aqueous periplasmic space sandwiched in between. The outer membrane (OM) is an asymmetric bilayer with lipopolysaccharide (LPS) on the outer leaflet and phospholipids on the inner leaflet. Porins on the OM aid passive diffusion of nutrients and antibiotics. The inner membrane (IM) is composed of phospholipids and contains transporters. A thin layer of peptidoglycan (PG) cell wall exists in the periplasm, which is anchored to the outer membrane by Bruan’s lipoprotein (Lpp). The periplasm is also rich in macromolecules including proteins and glycans. OPGs are special glucans with a glucose backbone and modifications such as phosphoethanolamine, phosphoglycerol, and succinyl residues. OPGs contribute to the net negative potential across the OM. The selective permeability of the outer membrane leads to the development of a membrane potential. The figure was created using BioRender.com.

In all living organisms, the membrane envelope defines the boundary of the cells and serves to protect the cellular content from the external environment. The composition of the cell envelope is different between Gram-positive and Gram-negative bacteria. In Gram-positive bacteria, there is a single cytoplasmic membrane and a distinct cell wall made of a thick peptidoglycan layer [1,2]. In contrast, Gram-negative bacteria contain a cytoplasmic or inner membrane (IM), a thin peptidoglycan layer, and an outer membrane (OM) (Figure 1). The OM constitutes an extra protection barrier, contributing to the high level of intrinsic multi-drug resistance in Gram-negative bacteria [3]. The lipid and protein composition of the OM and IM varies [4,5]. The IM is a typical phospholipid bilayer rich in phosphatidylethanolamine, phosphatidylglycerol, and cardiolipin [6,7]. Conversely, the OM is an atypical phospholipid bilayer with an external leaflet composed of a lipopolysaccharide (LPS) monolayer and an internal leaflet of a phospholipid monolayer, similar in lipid composition with the IM [8,9]. The OM is rich in β-barrel proteins such as porins, which has been the focus of several excellent reviews [4,10].

The structural composition of the membranes influences the permeability of compounds including nutrients and antibiotics [3,11]. The simple phospholipid composition of the IM allows the passive diffusion of neutral antibiotics [7]. The structural composition of the OM is more complicated, leading to more complex penetration behaviors. Porins are protein channels serving as portals for most small, charged, and hydrophilic molecules. Many nutrients essential for the survival of the bacteria fall into this category. Some porins such as LamB, YddB, FadL, and PgaA are specific for the transduction of certain nutrients, while others are non-specific including OmpA, OmpC, and OmpF [4,12,13]. The LPS in the outer leaflet of the OM contributes to the negative surface charges and participates in an electrostatic interaction with charged antibiotics. Since the polysaccharide branches of the LPS are hydrophilic, they serve as a barrier to limit the permeation of hydrophobic antibiotics [14,15].

The periplasm is a unique feature of Gram-negative bacteria and houses a rich collection of small molecule ions, proteins, glucans, and the peptidoglycan scaffold. These components play important roles in structural support, cell division, secretion, envelope stress responding, signaling, and mobility [16,17,18,19]. The peptidoglycan scaffold (about 1.5–10 nm) in Gram-negative bacteria normally contains one or two layers of the interconnected structure, which is about one-tenth the thickness of the cell wall in Gram-positive bacteria (about 20–80 nm) [20]. A component of the periplasm of particular interest in this review is the osmoregulated periplasmic glucans (OPGs). These special glucans play an important role in the generation of the Donnan potential (DP) across the OM [21,22]. Bohin et al. recently reviewed the biosynthesis pathway of OPGs, whose building blocks, glucose residues, and phospholipids, are recruited from the cytoplasm and the IM, respectively [16]. The OPGs possess multiple net negative charges and are trapped in the periplasm [21,22].

Another integrated component of the bacterial cellular envelope, which plays an important role in bacterial drug resistance, is the array of efflux transporters [23,24]. While in Gram-positive bacteria the efflux systems only need to transport toxins across the cytoplasmic membrane, in Gram-negative bacteria there are two layers of membranes to cross. Five families of bacterial drug efflux pumps have been reported and many excellent reviews have been published on this topic and recent examples include [25,26,27,28]. A better understanding of the factors affecting OM permeability can aid in the development of more effective antimicrobial therapeutics. This is a potential strategy to address the problem of multidrug resistance in Gram-negative bacteria.

## 2. Membrane Potential (MP) Regulates Antibiotic Transport across the OM of Gram-Negative Bacteria

The bacterial MP is an electrical potential generated across the cell membrane that plays a key role in regulating several physiological processes including flagellar motility, ATP synthesis, cell division, dynamic communication, membrane transport, pH homeostasis, and antibiotic resistance [29,30,31,32,33,34]. Several potentials, including electrical potential, asymmetry surface potential, Nernst potential, and DP, contribute to the MP maintained across the bacterial cell envelope [35]. Generally, MP is a potential that arises when charges are partitioned across a membrane. An electrical potential is a form of MP that involves the establishment of an ion gradient across a membrane. The ion gradient is maintained by ion channels and active transporters. The presence of phospholipid head groups and LPS on the membrane surface creates another form of MP known as asymmetry potential across the bacterial membrane. A balance maintained between a group of permeable charges on both sides of the membrane generates the Nernst potential.

DP is the primary focus of this review as it has been recognized as an important factor in antibiotics permeability through the OM in Gram-negative bacteria. The term “Donnan potential” was first described by Teorell et al. and measurement of this potential across the OM of Gram-negative bacteria, *E. coli* and *Salmonella typhimurium*, was first reported by Stock et al. [22,36]. The discovery of OPGs by Schulman and co-workers led to the measurement of DP across the OM of Gram-negative bacteria [37,38,39,40]. The field of study involving DP in bacterial cells has evolved progressively in the last 50 years. Studies in the first 25 years focused on understanding the adaptation to osmolarity and its influence on the DP across the OM and membrane permeability [22,41,42,43,44,45]. In addition, DP has been identified as a surface potential in Gram-positive bacteria involved in cell mobility and adaptation to pH changes during this period [46,47,48,49,50,51,52]. In the recent 25 years, research focus shifted to the involvement of DP in the cell surface behavior in response to different environmental stimuli including charge, ionic strength, and pH [53,54,55,56,57,58,59,60]. In addition, the role of DP on compound accumulation in bacterial cells attracted research interest, in a bid to solve the fast-evolving problem of antibiotic resistance in bacteria [61,62,63,64].

DP arises from an imbalance in charges that result from small permeable ions crossing the OM freely in the presence of large impermeable charged molecules trapped in the periplasm. No active pumps are needed to maintain this potential, which is negative on the periplasmic side and normally contributes approximately 40 to 80 mV to the overall MP across the OM [22,35,42,59,65]. This net negative potential acts as a driving force to pull in positively charged antibiotics across the OM. This voltage is less than the estimated 150–200 mV MP across the IM of *E. coli* [56].

### 2.1. OPGs Contribute to the Generation of DP

OPGs in Gram-negative bacteria are acyclic and contain 6–12 glucose units linked by the β-1,2 glycosidic bonds in the parent chain and β-1,6 glycosidic bonds in the branches [66,67,68,69]. Most OPGs are decorated with charged functional groups such as Sn-phosphoglycerol, phosphoethanolamine, and O-succinate [70], thus on average OPGs carry a net negative charge of −5 per molecule [37,67]. OPGs are trapped in the periplasmic space after biosynthesis, due to their hydrophilicity and large molecular weight of approximately 2.5 kDa [21,22]. The charged OPGs contribute to an excess of negative charges in the periplasm of most Gram-negative bacterial cells [21,22]. In this negatively charged form, OPGs can act as electrostatic counterions and concentrate cations in the periplasm [56]. For example, in a recent molecular simulation study on the behavior of antibiotic polymyxin B1 in the periplasm, OPGs have been found to be a major interactor that forms prevalent interaction with the drug [71].

OPGs are maximally synthesized when bacterial cells are grown in hypoosmotic media and minimally synthesized when grown in hyperosmotic media [21,42,72,73,74,75]. Lacroix and co-workers reported that *E. coli* cells grown in a medium of low osmolarity (100 mOsm) synthesized a high level of OPGs, making up approximately 5% of the total dry weight of the *E. coli* cells [75]. This amount dropped 10-fold to approximately 0.5% when the cells were grown in a media with a high osmolarity of 600 mOsm. A study by Martinez et al. showed that DP decreased from −47.9 to −3.5 mV in *E. coli* when the K^+^ concentration in the external media increased from 10 to 500 mM [57]. An increase of the external osmolarity could play two roles leading to the reduction of DP: the direct reduction due to high ionic strength and the indirect effect through reducing OPG production.

### 2.2. Variation of pH of the External Media Alters DP

The pH of the external medium is an important factor to be considered while studying compound permeation across the OM. It not only alters the charge state of the compound but also changes the MP. A pH value that maintains the net positive charge on the antibiotics while maximizes the negative DP across the OM would favor penetration through the OM. Cama et al. studied the effect of pH and voltage on the permeation of norfloxacin through proteoliposomes containing *E. coli* porin OmpF [76]. Their single channel electrophysiology assay, using planar lipid bilayers derived from the proteoliposomes, showed that when a voltage of −25 mV was applied across the membranes, approximately two molecules of norfloxacin entered through the porins per second at pH 5. This influx rate was reduced by half when measured at pH 7. A voltage of −50 mV led to a flux of five molecules of norfloxacin per second at pH 5, and this flux rate was reduced to one-fifth when measured at pH 7 [76]. Their result revealed the importance of the positive charge of norfloxacin to the rate of its influx through porins. Norfloxacin possesses two ionizable functional groups, with pKas of 6.34 and 8.75, respectively [77]. At pH 5 the compound is positively charged, while at pH 7 it forms a zwitterion with a net charge close to neutral.

### 2.3. Synergy between Porins and DP in Cation Selection and Cellular Accumulation

Porins are important components of the OM in Gram-negative bacteria. The most abundant porins in *E. coli* are OmpA, OmpF, and OmpC. While OmpF and OmpC are non-specific porins that are associated with passive diffusion of small molecules, OmpA is involved in the maintenance of membrane integrity [3,4]. Since DP and antibiotic permeability are the focus of this review, OmpC and OmpF are of special interest, which are both cation-selective and permeable to different substrates including many antibiotics.

OmpC and OmpF are structurally very similar and yet different in their conductivity. OmpC is more cation-selective than OmpF because it has a more negative interior, while OmpF is more permeable than OmpC under normal physiological conditions due to its larger pore size [78]. Kojima and Nikaido showed in their study that mutations of specific residues lining the translocation pathway of OmpC or an increase of the ionic strength of the external environment can make OmpC as permeable as OmpF [79]. Their result revealed that an increase of NaCl concentration from 0 to 0.3 M resulted in an elevated permeability of OmpC to ampicillin, benzylpenicillin, and lactose to levels similar to OmpF. In contrast, non-ionic osmolytes such as sucrose, sorbitol or polyethylene glycol had no impact on the permeability of either OmpC or OmpF.

Other than directly changing porin conductivity, the osmolarity of the external media also influences the expression levels of porins. OmpF is induced in bacteria under conditions of low osmolarity [41,80,81]. In contrast, OmpC, a so-called osmoporin, is induced under conditions of high osmolarity [82]. This opposing effect has been proposed to be an adaptive strategy to maximize nutrients uptake under different conditions [81]. Intriguingly, low osmolarity also induces increased biosynthesis of OPGs as discussed above. This suggests that synergy may exist between the functions of OPGs and the porins.

## 3. Impact of DP on Susceptibility to Antibiotics in Bacterial Infections

In the clinical setting, Gram-negative bacteria play key roles in the emergence of bacterial infections as shown in Table 1. Among these bacteria, *E. coli* and *P. aeruginosa* are the top causative agents for these infections. Additionally, two thirds of the six bacteria notorious for antibiotic resistance, termed ESKAPE, are Gram-negative [83,84]. The presence of the OM in the Gram-negative bacteria is a bi-factor contributor to antibiotic resistance based on the impairment of compound penetration and enhancement of compound loss through efflux pumps. In addition, the periplasmic space between the OM and IM houses potential binders and metabolic inactivators, that could alter antibiotic activity. This constitutes a barrier to the design of new antimicrobials to curb antibiotic resistance in Gram-negative bacteria. Most compounds effective against Gram-negative bacteria are potent against Gram-positive bacteria but the reverse is not the case [85]. A good drug with activity against Gram-negative bacteria should combine the ability to permeate the OM and IM as well as evade the efflux pumps [84]. Frustratingly, the entry rule is not that simple for Gram-negative bacteria when compared to Gram-positive bacteria because physicochemical properties that promote penetration across the OM of Gram-negative bacteria oppose diffusion across their IM [84,86]. For instance, small hydrophilic compounds are good OM penetrators via porins and poor substrates for efflux pumps but slow penetrators across the IM. On the contrary, relatively large lipophilic compounds are good IM penetrators but poor OM penetrators and good substrates to efflux pump systems. A balance would be amphiphilic compounds such as fluoroquinolones and tetracyclines with multiple protonation sites that can cross the OM as charged complexes and diffuse through the IM as neutral species [87]. Then, again, the presence of potential binders in the periplasm stalls their antibacterial activity especially for those with targets in the cytoplasm. Silver published an excellent review on Gram-negative entry for antibiotics [88].

The impact of DP on bacterial infections is indirect and occurs through two means, alteration of bacterial virulence and adaptation to changes in osmolarity.

### 3.1. Alteration of Bacterial Virulence

Disruption of OPGs biosynthesis have been shown to impact bacterial virulence, suggesting a role of DP in the adaptation of bacteria in the host system [73,122,123,124,125,126]. Mahajan-Miklos et al. reported a reduction in virulence when mice models were infected with *Pseudomonas aeruginosa* strain 36A4, which possessed mutation on the *opg*H-like locus [125]. Bhagwat et al. reported a decrease in virulence after 15 days when mice models were orally infected with *Salmonella typhimurium* opgGH mutant strains as compared to the wild type strain and the opgGH mutant complemented with plasmid pBK16 [73]. Bowe et al. constructed a bank of 330 independent mutants of *Salmonella typhimurium*, which were screened for loss of virulence in a mice model [127]. The infected mice were monitored for 28 days. Their result showed that CL288, a mutant strain with an insertion in a gene sequence homologous to *opg*B, had attenuated virulence in the mice model [128]. OpgB contributes to the anionic charge of OPGs which indicates that DP may contribute to the virulence of the bacteria.

### 3.2. Impact on Susceptibility of Bacteria to Antibiotics

OPGs are key factors in the adaptation of Gram-negative bacteria to osmotic changes in the external environment [73,75]. Under conditions of high osmolarity, OPG production is minimized. The decreased OPG level is associated with a reduction in DP as well as a loss of the ability to concentrate positively charged antibiotics in the periplasm. Therefore, the bacteria become less susceptible to these antibiotics [73]. Medeiros et al. reported the effect of varying ionic strength of different media on the susceptibility of three Gram-negative bacteria to gentamicin [117]. *Pseudonomas aeruginosa*, *Serratia marcescens*, and *E. coli* K12 were the representative strains. For bacteria grown in nutrient broth, the minimum inhibitory concentration (MIC) of gentamicin increased 130-fold (*P. aeruginosa*), 8-fold (*S. marcescens*), and 36-fold (*E. coli*), when the concentration of MgCl_2_ increased from 0 mM to 29 mM. Alteration of the ionic strength which involved NaCl gave a similar result as with MgCl_2_. The MIC of gentamicin against *P. aeruginosa* increased 67 folds when the concentration of NaCl increased from 2.7 mM to 174 mM.

## 4. Effect of DP on Membrane Permeability to Selected Antibiotics

The net charge and hydrophobicity of antibiotics play a pivotal role in their permeability across the membrane barrier. Hydrophobic antibiotics tend to permeate the membrane system via diffusion [129], while hydrophilic antibiotics permeate the OM of Gram-negative bacteria primarily via porins [13,129]. The rate at which antibiotics flux through the porins depends on their charge, hydrophobicity, conformation and size, as well as the pH of the external medium [130]. The influence of DP on the membrane permeability of several classes of antibiotics have been reported, including fluoroquinolones, tetracyclines, β-lactams, and trimethoprim. These antibiotics are clinically relevant alongside aminoglycosides and macrolides. Additionally, a common feature shared by these antibiotics is the presence of ionizable functional groups, and thus the equilibrium between the charged and uncharged states.

### 4.1. Fluoroquinolones

Fluoroquinolones are part of the quinolone family of antibiotics derived from the parent compound nalidixic acid [131,132,133]. Fluoroquinolones derived their name from the possession of a quinolone core structure and a fluorine modification on C6 of the quinolone backbone. Chemical modifications have led to several generations of fluoroquinolones with a broad spectrum of activity against both Gram-positive and Gram-negative bacteria [134]. Fluoroquinolones act to inhibit DNA topoisomerases with DNA gyrase as the primary target in Gram-negative bacteria [135,136,137,138].

**Figure 2:**
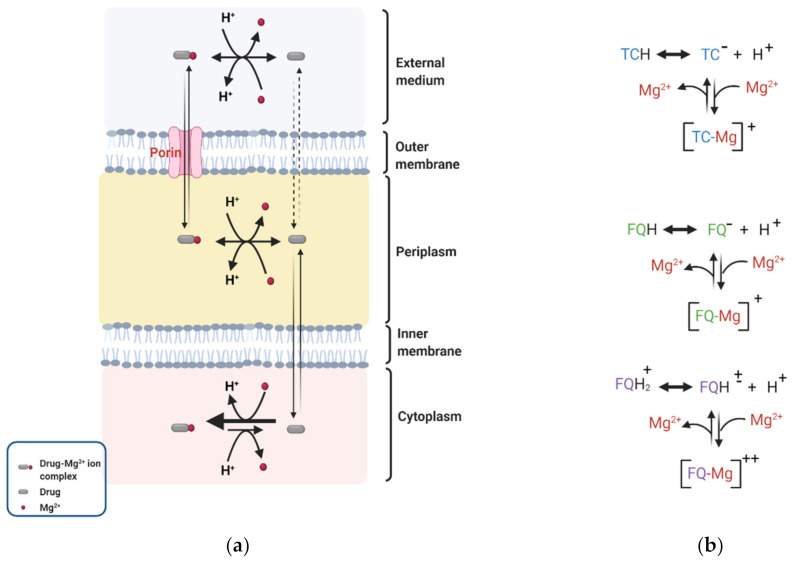
Donnan potential (DP) across the OM of Gram-negative bacteria drives the periplasmic accumulation of antibiotics such as tetracyclines (TC) and fluoroquinolones (FQ). (**a**) TC or FQ permeates the OM complexed with Mg^2+^, becomes protonated in the periplasmic and loses its Mg^2+^ to a neutral form and crosses into the cytoplasm, where it is deprotonated and complexed with Mg^2+^ again. (**b**) A notable difference between FQ and TC is that some zwitterionic fluoroquinolones [FQH]^±^ permeate the OM as fluoroquinolone-divalent ion complexes [FQ-Mg]^++^. Figures were created with BioRender.com.

Fluoroquinolones possess multiple protonation sites, which impact their permeability through the OM of the Gram-negative bacteria [65]. As an initial step to permeating the OM, fluoroquinolones chelate magnesium ions (Mg^2+^) associated with the LPS on the OM, conferring a net positive charge on these quinolones as illustrated in Figure 2 [139,140]. The potential sites for chelation on fluoroquinolones are the 4-ketone and 3-carboxylic acid groups [141,142]. Chelation of Mg^2+^ by fluoroquinolones leads to the formation of hydrophobic patches on the OM, an elevation of membrane fluidity, and subsequent enhancement of permeability [143]. Fluoroquinolones can permeate the lipid bilayer of the OM through porins or by the process of self-promoted uptake [140]. The more hydrophilic fluoroquinolones can diffuse through the cation-selective porins, mainly OmpF, into the periplasmic space [144,145]. The more hydrophobic fluoroquinolones enter through self-promoted uptake via the hydrophobic patches created on the OM.

DP has been shown to drive the periplasmic accumulation of fluoroquinolones [61,62,76]. Recently Prochnow et al. reported an approximately 2-fold higher concentration of ciprofloxacin accumulated in the periplasm of wild type *E. coli* cells compared to the external medium [62]. Pandeya et al. measured the subcellular accumulation of nine fluoroquinolones and revealed that the concentration of the fluoroquinolone accumulated in the periplasm of *E. coli* cells was approximately 4- to 18-fold higher than in the external medium [61]. The higher concentration in the periplasm compared to the external media suggests that factors exist across the OM to retain and accumulate fluoroquinolones. Lecomte et al. quantified the cellular uptake of norfloxacin, ciprofloxacin, pefloxacin and sparfloxacin in *E. coli* strains [146]. They observed that antibiotic uptake in the fluoroquinolone susceptible strain KL16 decreased by approximately 75 to 90% when the Mg^2+^/drug ratio was increased from 0 to 20,000 (wt/wt). A decrease in DP across the OM at the high Mg^2+^ concentration is likely a contributing factor to the reduction of accumulation. Once in the periplasm, fluoroquinolones dissociate from Mg^2+^ and an equilibrium is established between the charged and neutral fluoroquinolone species [65]. In the neutral state, these compounds permeate the IM to gain access into the cytoplasm (Figure 2).

### 4.2. Tetracyclines

Tetracyclines are broad-spectrum antibiotics with high affinities for ribosomes. They prevent the attachment of aminoacyl-tRNA to the ribosomal acceptor site and inhibit protein synthesis [147,148]. The first set of tetracyclines, chlortetracycline, and oxytetracycline, were products from natural sources *streptomyces aureofaciens* and *S. rimosus*, respectively [149,150]. Other compounds in the family are derivatives from either natural sources or semi-synthetic processes [151,152,153,154]. The mechanism of permeation of tetracyclines is highly similar to that of fluoroquinolones as illustrated in Figure 2a [65]. Most tetracyclines cross the OM via OmpF and OmpC porins, in magnesium bound forms [147,148]. A few derivatives with higher hydrophobicity may directly permeate the OM [155]. It was postulated that, unlike some fluoroquinolone-divalent ion complexes [FQ-Mg]^++^, tetracycline diffuses through OmpF and OmpC of susceptible *E. coli* strains as univalent ion complexes [TC-Mg]^+^ [87,156]. DP across the OM drives the accumulation of [TC-Mg]^+^ species in the periplasm.

Thanassi et al. showed that DP can drive the periplasmic accumulation of tetracycline [87]. The authors experimented with a porin single knockout *E. coli* strain (CM6; OmpF^+^ OmpC^−^) and a double porin knockout *E. coli* strain (CM7; OmpF^−^ OmpC^−^). Both strains were deficient in the Tet efflux pump. Their results showed an approximately 2-fold higher steady uptake of TC in CM6 compared to CM7. TC permeated the OM of CM6 quickly in the form of [TC-Mg]^+^ through OmpF but diffused slowly in the form of TCH across the OM of CM7. CM6 accumulated tetracycline at a concentration approximately 15-fold higher than the external concentration. The authors attributed a fraction of the whole cell accumulation in CM6 to the accumulation of [TC-Mg]^+^ in the periplasm driven by the DP across the OM. A 15-fold decrease in periplasmic [TC-Mg]^+^ concentration was observed when the Mg^2+^ concentration in the assay buffer was reduced from 1 to 0.03 mM. A high concentration of periplasmic [TC-Mg]^+^ resulted from the equilibration of both charged and uncharged species of TC across the OM. They suggested that DP may have concentrated Mg^2+^ in the periplasm and trapped these TC species as [TC-Mg]^+^ complexes in the periplasm. The contribution of DP to periplasmic [TC-Mg]^+^ accumulation was further demonstrated by the difference observed between TC accumulation values obtained from two analytical methods, filtration and centrifugation. The filtration method involves steps that promote leakage of charged species from the periplasm through OM, while the centrifugation method does not [157,158]. A reduction of 150 pmol/mg protein in TC accumulation level was obtained using the filtration method as compared to the centrifugation method, when the assay buffer was supplemented with 1 mM Mg^2+^. This difference was reduced to less than 50 pmol/mg protein when the assay buffer was supplemented with 0.03 mM Mg^2+^.

### 4.3. β-. Lactams

Penicillin was the first β-lactam discovered by Alexander Fleming [159]. The first semi-synthetic penicillin compound with therapeutic properties was developed later in 1940 [160]. Thereafter, other β-lactams such as cephalosporins were isolated from microbes and modified to generate semi-synthetic compounds [161]. The several generations of cephalosporins and their therapeutic effects were reviewed by Shahid et al. [162]. β-lactams are antibacterial that target the biosynthesis of the peptidoglycan cell wall by inhibiting the activity of transpeptidases [163,164,165]. These transpeptidases are examples of penicillin-binding proteins.

β-lactams permeate the OM of Gram-negative bacteria through porins [166]. The net charge of the beta-lactams is a key factor that influences the permeation of these compounds across the OM [167]. Bellido et al. assessed the permeation of several β-lactams across the OM of Enterobacter cloacae cells [168]. The negatively charged β-lactams, including cefotaxime, ceftriaxone, and carumonam, had low permeability coefficients (*p*-values). In contrast, positively charged cefepime and cefpirome had a 15-fold and 20-fold increase in *p*-values. They attributed the higher permeability of the net positively charged cephalosporins to the presence of cation-selective porins and the DP across the OM that promoted the periplasmic accumulation of the positively charged β-lactams. In another study by Sen et al., the apparent permeability coefficient of three cephalosporins across the OM of *E. coli* cells was measured at different ionic strengths [42]. The permeability coefficient of cephaloridine, a zwitterion, decreased from 6.5 to 5.2 μm/s with an increase in NaCl concentration from 0 to 0.25 M, which correlates to a decrease in DP from 100 to 5 mV. Reverse behaviors were observed for cefazolin (mono-anion) and SCE-20 (di-anion). When NaCl concentration increased from 0 to 0.1 M, their permeability coefficients increased from 0.1 to 0.48 µm/s and 0.005 to 0.048 µm/s, respectively. Their results showed a clear influence of DP on the permeability of these cephalosporins.

### 4.4. Trimethoprims

Trimethoprims (TMP) are broad-spectrum synthetic antimicrobial agents, which have a synergistic effect with sulphonamides [169]. TMP acts to inhibit the cytoplasmic target dihydrofolate reductase [170]. Phetsang et al. assessed the cellular accumulation of a trimethoprim probe (compound 12b) in wild type (ATCC 25922) and an efflux deficient *E. coli* strain (*∆tolC*), both in the presence and absence of CCCP. They found a 4- to 8-fold increase in the cellular accumulation of 12b in both the wild type and efflux pump deficient strains, compared to the external concentration [171]. In addition, cells treated with CCCP accumulated 8-fold more fluorescent trimethoprim compared to the untreated cells [171]. TMP probe 12b is small (<600 Da) and rich in amino groups, which would be positively charged under the experimental condition [172]. The authors proposed that cellular accumulation of 12b is due to entrance via porins and the DP across the OM [171].

## 5. Measurement of DP across the OM of Gram-Negative Bacteria

Direct and indirect methods have been used to measure the DP across the OM of Gram-negative bacteria [22,42,87]. A direct method involves the calculation of DP based on the distribution of radioactive monovalent ions such as ^22^Na^+^ and ^36^Cl^−^ between the external medium and the periplasm. The ratio of the distribution of these ions is inserted into the Nernst equation to obtain the DP. These ions are easy to work with since they diffuse freely across the OM but not the IM. Stock et al. quantified the DP across the OM of *Salmonella typhimurium* strain SL35 grown in M63 medium [22]. The estimation of DP was based on the distribution of [^22^Na^+^] NaCl or [^36^Cl^−^] NaCl between the external medium and the periplasm of the bacteria. The ratio of the ion distribution between these two compartments [j_periplasm_]/[j_external_], was inserted into the Nernst Equation (1) to obtain the DP across the OM. They observed that a decrease in NaCl concentration from 10 mM to 1 mM in the external medium resulted in an increase in DP from 17 mV to 31 mV and an increase in the estimated periplasmic osmotic strength from 209 mosM to 309 mosM. The distribution of [^22^Na^+^] was used for their calculation. Sen et al. modified the method for DP estimation previously reported by Stock et al. [22,42]. They used [^14^C] choline instead of ^22^Na^+^ [NaCl] or ^36^Cl^−^ [NaCl] to avoid high intensity radiation. They estimated the DP across the OM of *E. coli* HN455 strain grown in modified M63 medium, which contained 1 mM [^14^C] choline, 1 mM Imidazole and varying concentrations of NaCl. NaCl aided the transport of the radiolabeled choline into the cell. In HN455, the genes encoding the high-affinity choline transport system was disrupted to prevent cytosolic transport of choline. The ratio of [^14^C] choline distribution between the periplasm and external medium enabled the calculation of DP using the Nernst equation. Their result showed that DP across the OM of HN455 decreased from 96 mV to 7 mV as the concentration of NaCl in the modified M63 medium increased from 2 mM to 300 mM.

E = (59.2/z) log [j_periplasm_]/[j_external_] (1)

To minimize the usage of radioactive reagents, an indirect method of DP estimation has been developed. Sen et al. used quantification of OPGs as an indirect method to estimate the DP across the OM of *E. coli* K-12 (HN455 strain) [42]. They observed that OPGs reduced from 6.5% to 0.5% of the dry weight of bacteria cells as the ionic strength of the external medium increased from 2 mM to 300 mM NaCl. A correlation between the concentration of OPGs, periplasmic volume and DP was derived through computer simulation. They predicted that DP reduced from 117 mV to 4 mV as the ionic strength of the external medium increased from 2 mM to 300 mM NaCl through simulation, which is consistent with values from the direct measurement of DP as discussed earlier. Martinez et al. used a continuous affinity distribution method (FOCUS) optimization and a Donnan shell model to estimate the DP across the OM of *E. coli* K-12 strain AB264 [57]. Acid–base titrations were performed on intact *E. coli* cells at four ionic strengths (0.01, 0.05, 0.1 and 0.5 M KNO_3_) to determine the surface charge excess data. A plot of the charge excess data as a function of the bulk pH was used to determine the apparent dissociation constants. FOCUS was used to generate continuous pKa affinity spectra. The area under the Gaussian distribution for each pKa peak yielded different site densities, which were summed into the total binding site density (*L_T_*) values. In a modified Nernst Equation (2), the *L_T_* value, the charge (*z*), and the concentration (*c*) of the counterions in the background electrolyte, KNO_3_, were used to calculate the surface DP (*ψ_DON_*) of the bacteria. The methods used by the authors made it possible to observe changes in electrostatic parameters such as *ψ_DON_* when the concentration of K^+^ ions in the background electrolyte varied.

(2) $$\psi_{DON}=\frac{R.T}{z.\;F}\mathrm{arcsinh}\frac{\alpha_{D}.z_{g}\left(L_{T}\right)}{2zc}$$

## 6. Concluding Remarks

The permeation, distribution, and accumulation of charged antibiotics depend on the synergy among the pH of the external medium, DP across the OM, and the pH gradient across the IM. This synergy is pronounced when net positively charged antibiotics are involved. The pH of the external medium affects the net charge of the antibiotic, the expression of porins, and the size of the porin channels. DP is pivotal in driving the accumulation of charged compounds in the periplasm of Gram-negative bacteria. The type and magnitude of the net charge on the antibiotic dictates how much of the antibiotic will accumulate within the periplasm. Understanding the roles played by DP in drug accumulation is important in our effort to develop better penetrators that effectively breach the Gram-negative cell envelope.

## Figures and Tables

**Table 1:** **Major** Gram-negative bacteria pathogens as causative agents of bacterial infections.

Diseases/Conditions	Associated Gram-Negative Bacteria
Brucellosis [89,90]	*Brucella* spp.
Cholera [91,92]	*Vibrio cholerae*
Gastroenteritis [93,94,95]	*E. coli*, *Salmonella* spp., *Shigella* spp., *Campylobacter jejuni*
Gonorrhea [96,97]	*Neisseria gonorrhoeae*
Legionnaires’ Disease [98,99]	*Legionella pneumophila*
Pertussis [100,101]	*Bordetella pertussis*
Plague [102,103]	*Yersinia pestis*
Respiratory tract infection, Pneumonia [104,105,106]	*E. coli*, *Klebsiella pneumonia*, *Acinetobacter baumanii*, *Pseudomonas aeruginosa*
Shigellosis [107,108,109]	*Shigella* spp.
Surgical wound infection [110,111,112]	*E. coli*, *Klebsiella pneumonia*, *Acinetobacter baumanii*, *Pseudomonas aeruginosa*
Tularemia [113,114]	*Francisella tularensis*
Typhoid fever [115,116]	*Salmonella typhimurium*
Urinary tract and urinary-catheter infections [117,118,119,120,121]	*E. coli*, *Klebsiella pneumonia*

## Data Availability

Not applicable.
